# The practice of ‘doing’ evaluation: lessons learned from nine complex intervention trials in action

**DOI:** 10.1186/1748-5908-9-75

**Published:** 2014-06-17

**Authors:** Joanna Reynolds, Deborah DiLiberto, Lindsay Mangham-Jefferies, Evelyn K Ansah, Sham Lal, Hilda Mbakilwa, Katia Bruxvoort, Jayne Webster, Lasse S Vestergaard, Shunmay Yeung, Toby Leslie, Eleanor Hutchinson, Hugh Reyburn, David G Lalloo, David Schellenberg, Bonnie Cundill, Sarah G Staedke, Virginia Wiseman, Catherine Goodman, Clare IR Chandler

**Affiliations:** 1 Department of Social and Environmental Health Research, London School of Hygiene & Tropical Medicine, 15-17 Tavistock Place, London WC1H 9SH, UK; 2 Department of Medical Statistics, London School of Hygiene & Tropical Medicine, Keppel St, London WC1E 7HT, UK; 3 Department of Global Health and Development, London School of Hygiene & Tropical Medicine, 15-17 Tavistock Place, London WC1H 9SH, UK; 4 Dangme West District Health Directorate, Ghana Health Service, PO Box DD1, Dodowa, Ghana; 5 Disease Control Department, London School of Hygiene & Tropical Medicine, Keppel St, London WC1E 7HT, UK; 6 Joint Malaria Programme, Moshi, Tanzania; 7 Centre for Medical Parasitology at Department of International Health, Immunology and Microbiology, University of Copenhagen, Copenhagen K, Denmark; 8 Department of Infectious Diseases, Copenhagen University Hospital, Copenhagen K, Denmark; 9 Department of Clinical Sciences, Liverpool School of Tropical Medicine, Pembroke Place, Liverpool L3 5QA, UK; 10 Department of Infectious Disease Epidemiology, London School of Hygiene & Tropical Medicine, Keppel St, London WC1E 7HT, UK; 11 Department of Clinical Research, London School of Hygiene & Tropical Medicine, Keppel St, London WC1E 7HT, UK; 12 Department of Public Health and Community Medicine, University of New South Wales, Kensington, NSW 2033, Australia

**Keywords:** Complex interventions, Evaluation, Behavioural interventions, Health service, Low-income setting, Reflection, Trials

## Abstract

**Background:**

There is increasing recognition among trialists of the challenges in understanding how particular ‘real-life’ contexts influence the delivery and receipt of complex health interventions. Evaluations of interventions to change health worker and/or patient behaviours in health service settings exemplify these challenges. When interpreting evaluation data, deviation from intended intervention implementation is accounted for through process evaluations of fidelity, reach, and intensity. However, no such systematic approach has been proposed to account for the way evaluation activities may deviate in practice from assumptions made when data are interpreted.

**Methods:**

A collective case study was conducted to explore experiences of undertaking evaluation activities in the real-life contexts of nine complex intervention trials seeking to improve appropriate diagnosis and treatment of malaria in varied health service settings. Multiple sources of data were used, including in-depth interviews with investigators, participant-observation of studies, and rounds of discussion and reflection.

**Results and discussion:**

From our experiences of the realities of conducting these evaluations, we identified six key ‘lessons learned’ about ways to become aware of and manage aspects of the fabric of trials involving the interface of researchers, fieldworkers, participants and data collection tools that may affect the intended production of data and interpretation of findings. These lessons included: foster a shared understanding across the study team of how individual practices contribute to the study goals; promote and facilitate within-team communications for ongoing reflection on the progress of the evaluation; establish processes for ongoing collaboration and dialogue between sub-study teams; the importance of a field research coordinator bridging everyday project management with scientific oversight; collect and review reflective field notes on the progress of the evaluation to aid interpretation of outcomes; and these approaches should help the identification of and reflection on possible overlaps between the evaluation and intervention.

**Conclusion:**

The lessons we have drawn point to the principle of reflexivity that, we argue, needs to become part of standard practice in the conduct of evaluations of complex interventions to promote more meaningful interpretations of the effects of an intervention and to better inform future implementation and decision-making.

## Background

Increasing attention has been paid to understanding ‘what works’, for whom and under what circumstances in order for evaluations of health and health service interventions to be useful in informing wider implementation [1,2]. There is a growing body of literature on evaluations of complex interventions defined as interventions with multiple, interacting components [3-6], such as behavioural interventions in health service settings which may have several dimensions of complexity and include subjectively-measure outcomes *e.g.,*[7]. Within this literature there has been particular focus on the most appropriate research designs through which to evaluate these types of complex interventions [8,9], with guidance on selecting and measuring outcomes [5,6] and on evaluating the implementation processes and the influence of context on the delivery of an intervention, and on its effect [10,11]. Through our experiences of evaluating complex behavioural interventions in ‘real-life’, low-income, health service settings, we have become aware of the importance for validity of data of the dynamics at the interface of researchers, fieldworkers, participants, and data collection tools that form the fabric of the evaluation components of trials. We have been unable to identify guidance or any systematic approach to being alert to and managing issues arising at these interfaces during the enactment of evaluation activities that may influence how emerging data can be interpreted.

For research conducted in ‘real-life’ settings, it cannot be assumed that the delivery of a complex intervention or its evaluation will be exactly as planned or intended in the design stage of a trial. Literature on process evaluation highlights the importance of taking a systematic approach to documenting and accounting for this deviation, reporting the actual implementation, receipt and setting of an intervention in order to interpret its effects [12,13]. Within this literature, it is acknowledged that understanding the dynamics between the trial context and the nature of the intervention is important for interpreting the mechanisms of effect of an intervention and its potential transferability [14]. However, as the formal objective of process evaluation is to investigate the delivery of the intervention [15], this research practice does not typically extend to considering (or reporting on) the dynamics between the trial context and evaluation activities, and their potential implications for interpreting trial data. A number of studies have explored and reported on particular aspects of the delivery of the evaluation of an intervention, for example the recruitment and consent procedures of a trial [16], or major adverse events arising which led to the discontinuation of a trial arm [17]. While such examples offer a snapshot of processes and interactions that may occur during an evaluation, they fall short of proposing a systematic approach to becoming aware of and managing the dynamics of data generation through the whole process of conducting evaluation activities in real-life contexts, as has been adopted in process evaluations of intervention delivery [14].

**Figure 1:**
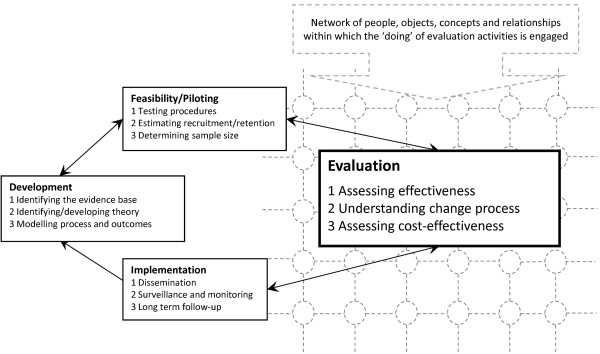
**Focus on ‘doing’ evaluation.** Adapted from Medical Research Council [4], this diagram shows the stages of the process of a complex intervention, highlighting the stage of ‘doing’ evaluation activities in a real-life setting, which is the focus of this paper.

We consider data generation in a trial to be a set of processes and influences that are embedded in a network of objects, people, concepts, goals and relationships. This network constitutes both the trial activities—the delivery of the intervention and evaluation—and also the context in which they are conducted [18]. Figure 1 draws on the key stages of the development, evaluation, and implementation of a complex intervention depicted in recent guidance from the Medical Research Council [4], and we highlight the evaluation stage, situated within this influencing network, or the ‘fabric’ of the trial in real life. Thus, we draw attention to the purpose of this paper: to consider the reality of ‘doing’ evaluation of an intervention and how it may contribute to interpretations of trial outcomes.

In this paper, we reflect on our own experiences of the need to respond to challenges arising during the execution of evaluation activities as part of a trial or similar research study of an intervention. Existing literature relating to evaluation practices focuses on project or trial management, research ethics and quality assurance. Trial management literature aims to ensure the efficient operationalization of a trial within budget and time constraints; *e.g.,* the *Clinical Trials Toolkit*[19]. Research ethics literature incorporates the standard codes upheld by ethics and institutional review boards as well as exploring how ethical practices and issues are negotiated in local trial contexts *e.g.,*[20,21]. Literature on quality assurance in trials has focused on internal validity through the standardisation of research processes [22,23], and promotes the use of independent boards to monitor progress of the trial and safety data against critical interim and endpoints [24]. However, a gap remains regarding the dynamics of conducting the evaluation component of trials in practice [25]. There is no cohesive guidance for researchers on how to consider the potential implications of real-time decisions made when enacting evaluation activities for the interpretation of trial results. In this paper, we will draw on our experiences of ‘doing’ evaluation in a research context to present lessons learned for negotiating the reality of evaluation and reflecting on the subsequent implications for interpreting trial outcomes.

## Methods

### Our research context

We draw on our experiences of conducting research-focused evaluations of interventions to improve malaria diagnosis and treatment in real-life health service settings, as part of the ACT Consortium (http://www.actconsortium.org). Nine ACT Consortium studies, in six countries in Africa and Asia, have used a range of methods to evaluate interventions that target health worker and/or patient behaviours in relation to the appropriate diagnosis and treatment of malaria. They are located in settings where overdiagnosis of malaria and unnecessary prescription of antimalarials are commonplace. This is typically underpinned by an entrenched practice of presumptive treatment of malaria by health workers, even when diagnostic facilities are available, and by a range of social influences including patient expectations when seeking care for febrile illness *e.g.*[26-28]. The interventions can be defined as complex [6]; they comprise multiple different and interacting components, require considerable shifts in behaviours by intervention recipients, involve a variety of outcome measures, and are implemented at various levels of low-resource health services, including public health facilities, community health workers and private drug vendors. See Table 1 for a summary of the studies represented in this paper.

**Table 1:** Summary of studies represented in this paper

**ACT Consortium study**^**1 **^**and location**	**Study aims**	**Evaluation activities conducted**
**1, Uganda**	Cluster randomised trial (CRT) to evaluate an intervention package to enhance health facility care for malaria and febrile illnesses in children.	1) Cross-sectional community surveys; 2) cohort study of children; 3) patient exit interviews; 4) health centre surveillance; 5) key informant in-depth interviews (IDIs) and questionnaires; 6) community focus group discussions (FGDs).
**2, Uganda**	CRT to evaluate the cost-effectiveness of artemisinin-based combination therapies (ACTs) following the introduction of rapid diagnostic tests (RDTs) for the home-management of malaria at the community level.	1) Blood slide readings to assess appropriateness of treatment; 2) follow-up household and morbidity surveys 3) FGDs and IDIs with community medicine distributors and community members.
**3, Uganda**	CRT to evaluate the impact of introduction of RDTs to drug shops on the improvement of rational drug use for case management of malaria.	1) Blood slide readings to assess appropriateness of treatment; 2) follow-up household surveys; 3) FGDs with drug vendors, carers and health workers; 4) adverse event surveillance.
**4, Tanzania**	Before-and-after observational evaluation of interventions to increase access to RDTs in public facilities and to ACTs in public and private facilities.	1) Household, health facility and outlet surveys; 2) post-intervention key informant interviews; 3) mixed qualitative methods including mapping exercises; rapid assessments of communities, IDIs and FGDs.
**5 (a), Cameroon**	CRT to evaluate basic and enhanced provider interventions to improve malaria diagnosis and appropriate use of ACTs in public and mission health facilities.	1) Intervention delivery evaluation (questionnaires, stocking records); 2) patient exit survey; 3) analysis of facility records and facility audit; 4) provider survey.
**5 (b), Nigeria**	CRT to evaluate provider & community interventions to improve malaria diagnosis using RDTs and appropriate use of ACTs in public health facilities and private sector medicine retailers.	1) Intervention delivery evaluation (questionnaires, stocking records, records of school-based intervention); 2) patient exit survey; 3) analysis of facility records and facility audit; 4) provider survey; 5) household survey.
**6, Afghanistan**	Individually randomised trial (IRT) and CRT evaluating an intervention to improve diagnosis and appropriate treatment of malaria with RDTs at health clinic level, and among community health workers.	1) Clinic based data collection; 2) entry and exit interviews with patients; 3) IDIs with health workers; 4) data collected from community health workers.
**9, Tanzania**	CRT evaluating health worker and patient oriented interventions to improve uptake of RDTs and adherence to results in primary health facilities.	1) Health facility data collection; 2) patient exit interviews; 3) intervention delivery evaluation (observations, questionnaires, IDIs); 4) follow-up household survey; 5) IDIs with health workers.
**15, Ghana**	IRT to evaluate an intervention to introduce RDTs to health facilities to improve diagnosis and appropriate treatment of malaria.	1) IDIs with health workers; 2) FGDs with community members. *Conducted* via *a separately funded project:* 3) blood slide reading to assess appropriateness of treatment; 4) health facility-based data collection; 5) follow-up household survey.

^1^See the ACT Consortium website, http://www.actconsortium.org, for more information on each of these studies.

Studies were designed to enable rigorous evaluation of the effects of interventions with the intention to inform policy and programmes in the future implementation of malaria diagnostics and treatment [27]. The design of the studies, therefore, had to balance needs for internal validity—an accurate representation of whether an intervention ‘works’ in a given setting—and external validity—the ability to generalise the results beyond a specific scenario, through careful evaluation designs [29]. Despite careful planning and piloting, we encountered a number of challenges and made a number of changes in the implementation of evaluation activities that we had to take into account in interpreting the data produced.

### Methodological approach

To elicit experiences and generate ‘lessons learned’, we used a collective case study design [30] based on an iterative, reflexive approach to study multiple cases of studies within their real-life contexts [31]. Each study in Table 1 was considered a case, unique in their combinations of research question, setting, and research team. Links existed between all cases through some investigators contributing to multiple studies and all being connected under the collaborative umbrella of the ACT Consortium. We sought to explore experiences of the implementation of our evaluation activities within three key domains: challenges and opportunities faced in the field when implementing evaluation activities; how these challenges/opportunities were negotiated (or not); and perceived impact of these challenges/opportunities and consequent actions taken.

We drew on multiple sources of information including in-depth interviews with investigators and study coordinators; rounds of discussion and reflection among those connected to the studies and those providing overarching scientific support across the ACT Consortium; informal participant-observation by JR, DD, and CIRC via engagement with studies as they were conducted; and reflection on study activities, documentation, and interpretation. The internal, embedded perspective of this set of methods enabled us to draw on our ‘institutional knowledge’ of the cases and their contexts in a way that would be extremely difficult for someone external to the studies. Our reflections were also supported by reviews of relevant literature to situate and interpret our experiences within a wider context of experimental research in low-income country settings. Formal analysis was conducted of the transcribed in-depth interviews using a framework approach [32], and this was used to provide an initial summary of experiences that formed the basis of further reflection, discussion and interpretation in multiple iterations between August 2012 and July 2013.

This research was conducted as an internal exercise within the ACT Consortium, involving only the authors and their reflection on their own experiences; as such, ethical approval was not sought beyond the original ethical approvals granted for each of the individual studies represented here.

## Results and discussion

### Lessons learned from implementing evaluation activities

We interpreted a set of first order constructs from across our collective experiences, and have categorised these as a set of ‘lessons learned’ from the implementation of evaluation activities and from our reflections on the potential implications of decisions made during the evaluation in response to contextual changes and influences. These influences included the networks of relationships, cultures, and expectations within which evaluation activities were conducted. Each lesson is described below with an overview, drawn from collective reflection and interpretation across the different cases, and with one or more specific examples from the cases and further interpretation through reference to existing literature. Although not every case contributed examples for every lesson, each lesson represents the interpretation of experiences from across multiple cases. A summary of the six lessons is presented in Table 2, and examples of experiences from across the different studies, which contributed to the identification of each lesson, are presented in Additional file 1.

**Table 2:** Summary of the lessons learned from our experiences of ‘doing’ evaluation

**Lesson learned**	**Summary of learning**
1. Different interpretations of study objectives and ‘success’ among team	Through pre-intervention and ongoing training, foster a shared understanding across the entire study team of why data are being collected, the processes and goals valued in the study and how individual practice feeds into the study’s rationale and outcomes.
2. Value of good communications to address challenges as they arise in the field	Plan intra-study communications structures carefully to ensure staff at all levels feel empowered to engage in reflection on the progress of the evaluation and interpretation of its outcomes, for example through frequent, supportive meetings and clear mechanisms for reporting and managing issues that arise.
3. Dialogue between different components of the evaluation	Establish mechanisms for ongoing collaboration between sub-study teams, to share experiences and observations from across study components, to encourage interpretation of research activities as the trial progresses, and to facilitate the synthesis of data from different disciplinary perspectives at the analysis stage.
4. Value of role of field research coordinator	Recognise, and support, the vital role of a field research coordinator in bridging the everyday, practical project management of a study, with an ongoing, scientific interpretation of evaluation activities, which can feed into generating meaningful results.
5. Value of collecting field notes during evaluation	Promote a continuous, inward reflection on the activities of an evaluation among team members through mechanisms for collecting, regularly reviewing and storing field notes, helping to make more meaningful interpretations of trial results at the analysis stage.
6. Recognition of, and reflection on, overlap between intervention and evaluation	In addition to careful planning and piloting of evaluation activities, the establishment, and maintenance, of the processes and structures described above should help the timely identification of and reflection on possible overlaps between intervention and evaluation activities, to feed into interpreting the trial results and usefully informing future implementation of the intervention.

### Training the study team to generate a shared understanding of objectives

Training of study staff, including field workers (the ‘field team’) and study coordinators, is a fundamental, and perhaps obvious, component of the planning and preparation for conducting evaluation activities as part of an intervention trial. Ensuring staff responsible for data collection, data management, and other activities at the ‘front-line’ of an evaluation are familiar with the study protocol and standard operating procedures (SOPs) is undeniably important. However, we should not assume that such training will necessarily translate to a shared understanding of the study objectives and research values as held by those with more scientific responsibility for the project. Particularly in evaluations of behavioural interventions, where outcomes are reported by participants or observed by field teams rather than objectively measured, a field worker’s understanding of the trial objectives will influence the way in which this data is elicited and recorded.

Although training of study teams was conducted prior to the commencement of evaluation activities in each of our projects, several scenarios arose which seemed to reflect differing interpretations of study objectives among study staff, particularly in what constituted ‘success’ of the project. Field teams sometimes appeared to equate success with active uptake and use of intervention technologies and ideas, suggesting that the intervention was ‘working’ as hoped or expected. By contrast, investigators saw success as the ability to evaluate whether (and why) an intervention was working and was taken up. In the cluster randomised trial in Afghanistan, trial staff reported giving corrective assistance to community health workers (CHWs) receiving the intervention, some of whom had conveyed during the data collection for evaluation that they had struggled to interpret correctly the result of the rapid diagnostic test (RDT) for malaria. Although a rare occurrence, when these difficulties were identified in the field trial staff felt it was important to provide additional advice to CHWs to improve their use of the RDT in line with the intended intervention and their diagnosis practices of malaria, thus potentially influencing the evaluation of the intervention’s success. The investigators recognised that this potentially compromised their research aim to evaluate the rollout of RDTs in the community in a ‘real-world’ setting in Afghanistan, where such supervision and feedback on CHWs’ practice were not commonplace. A decision was made not to restrict trial staff from advising CHWs, but to record and consider the likely effect of this at the analysis stage. In this example, the research aims of the trial were undermined by field staff’s concern for improving malaria diagnosis. Their activities, seen as ‘evaluation’ by the trial, in practice incorporated an additional ‘intervention’ activity. Good communication within the team, achieved through regular calls and close working relationships between the lead investigators and field staff, meant these additional activities could be known, recorded, and a decision made by the investigators to document such occasions.

It is important to remember that staff members responsible for enacting evaluation activities may hold different perspectives of a study’s objectives, which can influence the implementation of evaluation activities, despite the presence of, and training on, study protocols and SOPs [25]. In the Afghanistan example, these differing perspectives included the lead investigators’ concerns towards evaluating the intervention in the ‘real’ context into which it would likely be scaled-up and the field team’s concerns towards improving diagnosis and treatment of malaria in the local communities in which they were situated. The enactment of evaluation activities may thus reflect negotiations between different sets of objectives held among staff including the scientific trial objectives, personal objectives of being seen to do a ‘good job’ within the context of the study, and objectives towards the welfare of the groups of people directly engaged in the study.

The relationships between field staff, the trial, and the surrounding community have been explored in recent literature on research ethics ‘in practice’ *e.g.,*[21]. The process of producing high-quality data is a subjective, creative one, underpinned by particular values of what constitutes ‘dirty’ or ‘clean’ data [33,34]. As such, it is crucial to ensure that there is a shared understanding across the whole study team of the specific values held by those leading the research from a scientific perspective. Both content and timing of training can facilitate this. The content of training should extend beyond the practicalities of simply how to collect or manage data in line with SOPs, to an emphasis on *why* data are being collected, what processes and goals are valued within the project, and how staff members’ practices feed into these. Pre-trial training is required, but ongoing training, whether formal or more informal through regular supervision and feedback, will generate a greater understanding of a study’s rationale and objectives. Integrating this training within the day-to-day activities of staff will offer opportunities to address challenges they face, for example, negotiating personal and trial priorities. It should also help to ensure a heightened awareness among staff of their practices, and provide opportunities for reflection and dialogue within the study teams of how these challenges, negotiations, and practices influence the outcomes of a study.

### Promoting communications within the study team

The need for good communication between members of a study team is well recognised, and its role in the effective management of a research project is of little debate. It is important to consider in more detail the communication structures within a study and what information should be shared during the roll out of an evaluation. These elements may influence what study team members consider necessary to share about ongoing evaluation activities, their motivation to do so, and the subsequent actions taken. We recognised the value of having structures in place enabling the timely sharing of information about issues that arose while staff members were implementing evaluation activities, including contextual changes in the field, interactions between evaluation and intervention activities, and other unexpected events or complications. This helped bring awareness to the day-to-day progress of the evaluation and facilitated responsiveness of more senior staff to scenarios that had implications for meaningful interpretation of the data.

Investigators from several studies reflected on the value of a close communication system during evaluation, for example in Ghana (study 15), where regular meetings were held involving the field staff, study coordinators, senior investigators, and principal investigator. These meetings were considered to have enabled field staff to raise challenges they faced during evaluation, such as how to manage clinicians’ questions about the high number of negative malaria test results produced without greatly influencing the nature of the intervention received by participants. As a result, a decision was made for field workers to advise clinicians to continue to ‘do what they would normally do’, until after the period of data collection when feedback on the use and interpretation of diagnostic tests was given to the participating clinicians. In one study in Tanzania (study 4), investigators highlighted the importance of a responsive communication system during evaluation. One investigator described challenges faced in the field with meeting the sampling targets for the evaluation activities in some areas, due to reported contextual changes such as shifts in malaria control strategies and the epidemiology of fever in these areas. The investigator found it extremely valuable to have a system through which the problems field workers faced with recruitment, and the underlying contextual factors, could be communicated to her rapidly. This enabled her to seek advice from the study statistician and for ‘on-the-hoof sample size discussions’ to be held to inform quickly how recruitment activities in the field should progress, without delaying them. Another investigator from this study also highlighted the value of having a team leader who was able to communicate well with the field staff to detect any problems they were facing in their work, and was also empowered to communicate further up the chain to the study coordinators, to enable timely decisions to be made, and their scientific impact considered.

Members of field teams have been described previously as holding a difficult position with conflicting responsibilities and accountability both toward the research project and toward its ‘participants’, with whom they may have existing direct or indirect social relationships, obligations or expectations [35]. For example, field staff may feel obliged to extend services to the neighbours of a household randomly allocated to ‘receive’ evaluation activities such as testing (and subsequent treatment) for malaria. Barriers to field staff reporting challenges they face in negotiating field activities, including interactions with participants and the framing, phrasing, or ordering of questions or procedures, may reflect these social positions, as well as a lack of understanding of the potential (scientific) implications of such challenges for the study. This may be made more difficult by trial management structures; in all of our trial settings a strong hierarchical structure was apparent, reflecting the ‘command and control’ structure of health organizations in many African countries [36]. We found it necessary to try to counteract these hierarchies by encouraging dialogue ‘up’ to those with scientific responsibility for studies, together with demonstration that issues raised were taken seriously. Engagement of all staff in reflection on the approach to, and progress of, the evaluation and how the evaluation activities will feed into the results may provide a greater sense of satisfaction, particularly among lower level staff members, in their role in the research process [37]. Moreover, this may promote and legitimize a heightened perception of responsibility towards the study’s objectives, increase team reflexivity on their practices and how they contribute to the production of results, and contribute to increased rigour and quality of research activities [38]. We recommend careful planning of communication structures to include frequent meetings where engagement of staff at all levels is encouraged in a supportive, reflective environment, and clear mechanisms for reporting challenges faced, decision making, and giving prompt responses in decision making.

### Ongoing dialogue between different components of the evaluation

In addition to mechanisms to promote communication up and down the hierarchy of staff team members, it is valuable to consider the interaction between teams conducting different components of the evaluation and different sets of activities. Our evaluations consisted of multiple teams conducting different evaluation activities for quantitative, clinical, process, or qualitative outcomes. Projects typically intend to bring together results from different components at the end of the project. However, our experiences point to the value of dialogue during the trial, with benefits not only for interpreting the trial results, but also for informing decision-making to improve implementation of intervention and evaluation activities.

In one study in Uganda (study 1), a qualitative research team conducted process evaluation activities alongside ongoing intervention activities, with reflections and emerging findings being shared with the broader study team and principal investigators at regular meetings. An example of the value of this communication came from the ongoing analysis of in-depth interviews with health workers, who had received training as part of the intervention and who were asked to record a small amount of information in the health facility register in addition to routine data collected. The sharing of emerging findings from these interviews highlighted dissatisfaction among some health workers relating to the perceived extra burden of work created by involvement in the trial, and the reluctance of some to record data as requested without additional payments or benefits. The structures in place enabled the qualitative research team to communicate these issues in a timely way, resulting in discussion among the broader study team about how to address health workers’ concerns and the potential impact of any changes made on the interpretation of the intervention’s effect. It was decided to supply pens, sugar, and tea to all health facilities enrolled in the trial to recognise health workers’ involvement and support the ongoing work required to collect the additional health facility data. This decision was carefully noted for assessing the exact nature of the intervention as received and experienced by health workers, and for future interpretation of the trial results.

Literature exploring the different components of evaluations of complex interventions has tended to focus on the incorporation of these at the point of the final analysis of a trial, for example in terms of the value of process evaluation data for interpreting effects seen [13] or of the methodological and epistemological challenges of synthesizing multiple sources of data [10]. However, this approach overlooks the potential value of ongoing dialogue between the different strands of a study team and their activities as the evaluation is being conducted. Others have identified the benefits of building qualitative work early into the implementation of a trial to highlight challenges faced ‘in the field’ with conducting intervention and evaluation activities and to help their timely resolution [39,40]. Building on this, we recommend enabling effective team collaboration wherein experiences and observations from work done by different study components and team members from all levels feel engaged and encouraged to contribute their interpretation of research activities as the trial progresses. Combining interpretations from various disciplinary perspectives may help study teams to develop creative and appropriate responses to challenges faced, and promote ongoing reflection of their scientific implications. In addition, coordination between different groups during trial implementation may help to overcome any challenges faced at the point of analysis, in synthesizing multiple data sources from different disciplinary perspectives [41].

### The role of field research coordinator

Our collective experiences also pointed to the value of having in the project team one (or more) person(s) who takes responsibility for managing the day-to-day duties of evaluation activities as planned, but who also has the capacity (and time) to reflect on the activities in the field from a broader scientific perspective. This vital role should bridge the functions of overseeing coordination of activities and problem solving, understanding what is happening ‘on the ground’ in the context of the evaluation, and the ongoing reflection and interpretation of the potential impact on the study’s results. The position of the field research coordinator (or other, similar title such as ‘field manager’) would thus be able to align day-to-day project management with an in-depth understanding of the scientific implications of decisions made and be in a position to discuss this with the principal investigators, and contribute to the scientific oversight of the study including development of study protocols, evaluation activities, and analysis plans. This may be particularly valuable in settings where the principal investigators and/or scientific leads are situated away from the field site(s) for the study.

Across the studies represented here the role of field research coordinator was increasingly recognised as important as the projects entered and progressed through their life cycles. In our Ugandan projects (1, 2 and 3), a field research coordinator was located in or near to the main field sites in order to become aware of and address unexpected challenges or events arising in the enactment of evaluation activities on a day-to-day basis. It was considered important that this person had a thorough understanding and involvement in the scientific oversight of the project. This helped them relate every day issues encountered to the research objectives, recognising where changes or additions might be required in order to ensure data collected were meaningful, and helped the communication of difficulties or complications, and their potential solutions, to senior investigators. This was often a challenging role to play in terms of the levels of responsibility faced in both project and research management, but field research coordinators agreed that they were very well placed to understand in detail how activities played out in the field and to feed this into their contributions to the analysis of evaluation data and interpretations of results. In the Cameroon trial (study 5a), an investigator noted the absence of such a position in the qualitative study team as challenging in relation to maintaining staff members’ interest and understanding of the social science activities in the field. The field staff had had limited experience in social science due to local capacity constraints, and until the lack of field research coordinator for these activities was addressed, the team struggled to balance the demands of day-to-day project management with a broader level of thinking in relation to the overall research question. The investigators acknowledged this may have limited the scope, flexibility, and responsiveness of the social science activities in relation to ongoing reflections of the intervention as it was implemented, as the team were less likely and/or willing to explore beyond the original research questions and topic guide.

The need to build capacity in low-income settings for skilled research coordinators who can manage clinical trials with a scientific perspective in local settings has been previously identified [42]. In addition, the importance of the ‘hidden work’ required of a trial manager to establish and maintain trial processes within a clinical context has also been acknowledged [43]. We recommend that this position is recognised as playing a vital role in bridging the evaluation activity ‘in the field’ with the higher level interpretation of data and results, thus negotiating the practical and the scientific work of an evaluation in order to generate meaningful results. To achieve this, the field research coordinator should ideally be situated close to the study field sites, have an in-depth and ongoing understanding of the intervention and evaluation objectives and be provided with appropriate resources and support to both manage day-to-day research activities and reflect on them in light of the overall project.

### Keeping field notes

In addition to the established guidance and requirements for management of data in trials, for example the Good Clinical Practice guidelines [23], our experiences led us to consider the value of ongoing reflection and documentation of the evaluation in action. This is to capture contextual influences and changes that arise, decisions made in response, and reflections on these, to inform data analysis in the future. Process evaluation methods are valuable for documenting the contextual influences on the roll out of an intervention [14]; however, they do not routinely capture information on how those influences impacted on the rollout of evaluation activities, and thus the data collected. As discussed above, this reflection can occur through regular communications within and between research teams, ideally led by the field research coordinator or other project leads situated close to the field activities.

In the case of some of our studies, the time period of evaluation activities lasted for up to two years before formal analysis was conducted which hints at the potential challenges for trying to recall information about contextual influences at the point of interpretation of the data. For two studies in Uganda (2 and 3), the study coordinator actively recorded information in extensive field diaries throughout the period of conducting evaluation activities. He described making records following every trip to the field sites, interactions with the field teams, and noting difficulties or questions arising with data collection. An example issue recorded was dissatisfaction expressed by community medicine distributors (CMDs) in study 2 when their patients were followed up by study interviewers. Subsequent changes were made, for example conducting additional sensitization by the local study team to alleviate CMDs’ fears around their practice being ‘monitored.’ The coordinator anticipated the field diary would be particularly useful at the analysis stage for exploring reasons behind missing data from the evaluation data collection activities in study 3, for interpreting any patterns of reporting by drug shop vendors, to understand how the trial was being conducted and perceived by participants at that time. As such, he felt the field notes taken would be a valuable resource for reminding him of his ongoing reflections during the enactment of the evaluations, and feeding into interpretation of the results.

The ‘paramount importance’ of an audit trail for data management within a trial [44] has been emphasised through trial regulations and guidance [23]. However, framings of the audit trail in this literature tend to focus on changes to SOPs, data collection forms, and/or databases *e.g.,*[45]. We recommend the use of field notes to promote a continuous, inward reflection on the activity of an evaluation, to facilitate meaningful interpretation of the trial results. In addition, regular reviewing of field notes during the period of evaluation may help identify problems or questions that can be addressed in real time as they arise. This approach extends the focus of a process evaluation to documenting the reality of conducting the evaluation (in addition to the intervention), and echoes the reflexive perspective adopted within anthropological methods in the use of ethnographic field notes *e.g.,*[46]. The continuous recording and contemplation of the data collection process facilitates identification of the influences on the data collection process, including the subjective role played by the researcher (and research team) [47]. Hence, we recommend that mechanisms be built into the process of conducting evaluations to encourage study team members to capture their day-to-day experiences of evaluation activities, and to facilitate the regular reviewing of these notes and the systematic linking of them to the evaluation activities at the data interpretation stage.

### Addressing overlap between intervention and evaluation

The final lesson learned from our experiences relates to the identification and management of overlaps between evaluation and intervention activities in the field, and the consideration of potential consequences of this for interpreting the results of the trial. Careful planning and piloting of protocols and procedures should help to limit the possibility of evaluation activities interacting with the intervention, for example through separating the timing of conducting intervention and evaluation activities. However, from the perspective of the participants, both intervention and evaluation activities may be experienced and interpreted as the ‘intervention’. The ‘Hawthorne effect’, postulates that behaviour may be changed through awareness of being watched or evaluated. Studies can take this into account in design as something to be minimized and/or accounted for in analysis [48]. In addition, asking questions of participants, for example in process evaluation activities, may be interpreted as an intervention, with impacts on how individuals think, perceive the programme, and ‘perform’ trial outcomes [40]. Studies may, to some extent, be able to control for these effects by ensuring consistent activities in both intervention and control arms in a controlled trial design. Our experience suggests, however, the need to consider also a possible effect of evaluation activities changing or modifying the nature of the intervention itself, the perception of what constitutes the intervention by the recipients, and the intended mechanisms of change through which outcomes are realized. There are no easily identifiable guidelines on methods to identify and accommodate such interactions as they occur during a trial in action.

In one study in Tanzania (9), field staff conducting the evaluation of various components of an intervention to support health workers’ uptake of and adherence to RDTs for malaria were required to attend participating health facilities every six weeks to collect health worker-completed data forms and to monitor RDT stock levels. An investigator from this study described how they restricted these visits to a few specific tasks to minimize the impact on the health workers and their practice, and tried to conduct these activities similarly in both the intervention and control arms. Although from the perspective of the investigators and the study, these visits were ‘evaluation activities’, health workers could have interpreted interactions with field staff as ‘supervision’. This carried potential for a more pronounced Hawthorne effect for those in the intervention arms as they may have been more attuned to the behaviour seen as ‘appropriate’ by evaluators. These concerns about the possible interaction between evaluation activities and the intervention were echoed by investigators in another study in Uganda (1), where concern was expressed that visits to health facilities by field staff to collect surveillance data could be perceived as supervision, with potential for impact on practice in a context where supervision was infrequent. Investigators reflected on the difficulties of identifying, and deciding to what extent, and how to accommodate the possible effects of these activities on the nature of the intervention they were evaluating.

A challenge for these projects was to know exactly what the ‘intervention’ was from the perspective of recipients. This has implications for generalisability, to inform future scale-up or implementation of the intervention in other settings. While steps can be taken to anticipate and minimize overlaps between evaluation and intervention activities, our experience suggests that it is (almost) impossible to plan for all interactions that may occur when a trial is being conducted in the field. We recommend building into a trial a set of mechanisms to facilitate the identification and reflection on overlaps as they arise, and suggest that the actions and processes described in the lessons learned above would be instrumental for achieving this.

## Conclusion

In order to inform appropriate and effective implementation and scale-up of health and health service interventions, evaluations need to be useful and reflect the reality of the trial context. Just as interventions may not be implemented as planned, the ‘doing’, or enactment of evaluation activities may not be aligned in real-life with intentions and assumptions made in the planning stage. Changes arise, challenges are faced, and decisions are made, which all form part of the process of producing data for analysis and interpretation of the intervention [49]. The outcomes of complex behavioural interventions are typically subjectively measured and therefore their evaluation needs to be understood as an interpretive process, subject to the varying influences of the actors, activities and contexts that are engaged in an evaluation.

Our experiences of conducting evaluations of complex interventions in low-income country settings included negotiating a variety of day-to-day challenges that arose in the ‘doing’ of evaluation during the trial in action, and which reflected the specific networks of people, objects, relationships, and concepts in which trials operated. These issues could not easily have been predicted during the planning or piloting phases of our studies, and required a number of supporting structures (*e.g.*, mechanisms for communicating, documenting and reflecting on the reality of the evaluation) in order to ensure data collected would be meaningful for the interpretation of the trial outcomes and informing decisions on scale-up of an intervention. As a result of reflection on these experiences, we propose a set of ‘lessons learned’ that could be implemented systematically to improve future evaluation practice; a summary of these is presented in Table 2. At the core of our recommendations is the promotion of an ongoing, reflexive consideration of how the reality of enacting evaluation activities can impact the meaningful interpretation of trial results, thus enhancing the understanding of the research problem [50].

Reflexivity has been recognised as an ongoing, critical ‘conversation’ about experiences as they occur, and which calls into question what is known during the research process, and how it has come to be known [51]. The aim of reflexivity, then, is to reveal the sets of personal perspectives, interactions, and broader socio-political contexts that shape the research process and the construction of meaning [52]. Initial calls have been made for investigators to adopt a reflexive approach to reporting challenges of and changes to trials due to contextual factors affecting intervention delivery that may impact on the interpretation of internal and external validity [11,53,54]. Wells *et al.* recommend adaptation of the CONSORT reporting guidelines to support reflexive acknowledgment of how investigators’ motivations, personal experiences, and knowledge influence their approach to the delivery of an intervention in a research context, to better inform clinical and policy decision-making [11]. We argue for an extension of this perspective, centred on the intervention delivery, to reflexive consideration of the process of conducting evaluation activities in a trial. The ‘complexity and idiosyncratic nature’ of a trial [11], p14 does not affect only the delivery of the intervention, but the delivery of the evaluation activities also, and thus a reflexive approach would encourage greater awareness of the processes involved in enacting the protocol for an evaluation in a real-life context, and support more detailed reporting of these processes to aid decision-making. A reflexive approach could also facilitate a systematic consideration of the entirety of the evaluation and its activities, not just discrete stages of the trial such as recruitment or specific aspects such as ethics, quality assurance, or project management, as has been seen previously. Acknowledgment that conducting an evaluation is never as straightforward as a (comparatively) simplistic protocol would suggest on paper, and helping research staff members to reflect on their own role in the negotiations and nuances of the trial in action can lead to more informed and useful interpretations of the evaluation outcomes.

The events and questions that arose during our evaluations are unlikely to be unique to the trials of complex interventions alone, but familiar to those conducting other types of health intervention research. Additionally, we acknowledge that our experiences may not be representative of all other trials of complex interventions, either in low-income settings or beyond. We propose that the general principles behind our lessons learned could be valuable for other investigators evaluating interventions and/or conducting operational research, and balancing the demands for both internal and external validity of their trial. Rather than being seen as an ‘additional activity’, we recommend that this reflexive perspective be embedded within what is considered ‘good practice’ for the everyday conduct of a trial evaluating a complex intervention. We acknowledge that it will require efforts to create time and space to think about the progress of a trial, and that within a typical trial culture of working against the clock, this could prove challenging for some research teams. However, we suggest that taking this time will make for better practice in the long term, and that with increased practice, a reflexive perspective will become easier and more established in the trials of public health interventions. Extending the perspective offered in process evaluation approaches to consider the role of conducting evaluation activities themselves in the production of trial results, will surely increase understanding of what works and under what conditions [1,55], thus better informing effective scale-up and implementation of interventions.

## Abbreviations

ACT: Artemisinin-based combination therapy; CHW: Community health worker; CMD: Community medicine distributor; CRT: Cluster randomised trial; DSV: Drug shop vendor; FGD: Focus group discussion; IDI: In-depth interview; IRT: Individually randomised trial; RDT: Rapid diagnostic test (for malaria); SOP: Standard operating procedure.

## Competing interests

The authors declare that they have no competing interests.

## Authors’ contributions

JR, DD, BC, and CIRC conceived of and designed the approach to addressing this topic. JR, DD and CIRC coordinated the discussion and synthesis of experiences. LMJ, EKA, SL, HM, KB, TL, HR, SGS, VW, and CG participated in in-depth interviews and JR, DD, LMJ, EKA, SL, JW, LSV, SY, TL, EH, HR, DGL, DS, BC, SGS, VW, CG, and CIRC all contributed to rounds of discussion and interpretation of experiences. All authors contributed to the drafting and/or critical review of the manuscript, and all authors read and approved the final manuscript.

## Authors’ information

DD, LMJ, SL, KB, BC, TL, EH, SY, HR, JW, DS, SGS, VW, CG, and CIRC are all members of the London School of Hygiene & Tropical Medicine Malaria Centre.

## Supplementary Material

Additional file 1 A summary of the first order constructs interpreted from the data: examples of experiences across the different ACT Consortium studies which contributed to the identification of ‘lessons learned’. Click here for file
