# Supplementary material for: The practice of ‘doing’ evaluation: lessons learned from nine complex intervention trials in action
Source: Implement Sci. 2014 Jun 17;9:75. doi: 10.1186/1748-5908-9-75 (PMC4079170; doi:10.1186/1748-5908-9-75)
Supplement: Additional file 1 — A summary of the first order constructs interpreted from the data: examples of experiences across the different ACT Consortium studies which contributed to the identification of ‘lessons learned’. [file 1748-5908-9-75-S1.pdf]

Additional File 1: A summary of the first order constructs interpreted from the data: examples of experiences across the different ACT Consortium studies which contributed to the identification of 'lessons learned'

Study[1]	Lessons Learned					
	**Different interpretations of study objectives and 'success' among team**	**Value of good communications to address challenges as they arise in the field**	**Dialogue between different components of evaluation**	**Value of role of field research coordinator, with scientific oversight and project management duties**	**Value of collecting field notes during evaluation**	**Recognition of and reflection on overlap between intervention and evaluation**
**1**	Research team members expressed concern that data they elicited would show that the project 'failed' and had to be reminded that success of the project did not mean the intervention needed to be successful.	Discussion within team of issue faced by fieldworkers of whether to provide care to ill children in a household beyond the children recruited to participate in the survey.	Dialogue between qualitative and clinical trial teams to discuss health workers' dissatisfaction with extra work involved in trial participation and the need to recognise health worker contributions to the study.	Field research coordinator was involved with development of the study protocol and data collection tools alongside planning field logistics, ensuring that data collection procedures were logistically feasible in the field, were integrated with existing and planned databases, and would satisfy scientific objectives.		Health centres were visited regularly to collect and check study surveillance data, which health workers may have interpreted as a 'supervision' activity, and part of the intervention, rather than an evaluation activity.
**2**		CMDs[2] had concerns over researchers' follow-up of their patients; issues were discussed within the field team to address concerns.	Ongoing exchange of information between self-formed groups of CMDs (participants), field implementers and senior investigators, discussing completion of data collection forms and CMDs' experiences in the field.	Field research coordinator proved valuable for recording and reflecting on ongoing decisions made in the field for their practical and scientific implications.	Notes taken considered useful for interpreting changes and decisions made during fieldwork, and for interpreting reasons behind missing data.	Concerns arose about researchers' presence in the field being interpreted as 'monitoring' of CMDs, resulting in decision to reduce visits to study sites to once per month.
**3**	Some field workers were keen to demonstrate that intervention 'worked', rather than to evaluate its effectiveness; they felt 'challenged' if asked about		Discussion with qualitative field team highlighted ongoing dynamics of DSVs[3] recruiting patients into the study and how they managed the consent	*As for project 2 – same field coordinator.*	*As for project 2 – same field coordinator.*	Challenge of balancing understanding in detail how DSVs were enacting the intervention without too much scrutiny that could be interpreted as a

Study[1]	Lessons Learned					
	Different interpretations of study objectives and 'success' among team	Value of good communications to address challenges as they arise in the field	Dialogue between different components of evaluation	Value of role of field research coordinator, with scientific oversight and project management duties	Value of collecting field notes during evaluation	Recognition of and reflection on overlap between intervention and evaluation
	aspects of the data.		process.			'supervision' activity.
4	Different understandings among field staff of objective of processes for recruiting a representative sample of eligible patients in each clinic, resulting in isolated incidents of too few or too many patients recruited on one day.	Value of arrangements for flexible and responsive interaction between fieldworkers, field coordinators and study PI[4] in resolving unexpected issues as they arose in the field.		Field research coordinator considered very valuable for communication between senior investigators and field team, and for knowing when to communicate issues arising in the field to senior investigators.	Field notes used to capture exceptional circumstances faced in the field, any errors or changes to the processes of data collection.	
5a (Cam.)	Differing perceptions between laboratory/ clinical field staff and senior investigators of the value of (qualitative) social science activities for interpreting study outcomes, resulting in limited scope and depth of qualitative data collection at first.	Dialogue between field workers and senior investigators helped identify how some health workers were 'rationing' the use of RDTs[5] within the project, and contrary to the design of the intervention.		At first the lack of an in-field research coordinator for social science activities limited the scope and depth of data collection, but was later resolved by assigning an individual to this role.	Keeping ongoing records and notes on the supplies and stocking patterns of RDTs was very helpful for understanding how roll out of intervention was conducted, and differences between this and intended approach.	
5b (Nig.)	Some difference in interpretation of recruitment objectives and practices among field workers.		Process evaluation data indicated a shortfall between the numbers invited to training and the numbers trained, and a second round of provider training workshops were held.		Ongoing note-taking on events occurring in country including health worker strikes, was very important for informing the analytical approach and how to account for participants who didn't attend training sessions.	Slight modifications to the health management information system to collect extra data for the evaluation may have impacted on health workers' practice. Patient exit interviews by field team were seen as unwelcome supervision by some private providers

Study[1]	Lessons Learned					
	**Different interpretations of study objectives and 'success' among team**	**Value of good communications to address challenges as they arise in the field**	**Dialogue between different components of evaluation**	**Value of role of field research coordinator, with scientific oversight and project management duties**	**Value of collecting field notes during evaluation**	**Recognition of and reflection on overlap between intervention and evaluation**
						included in the study.
**6**	Field workers felt it was important to advise CHWs[6] on using RDTs where errors in practice were observed, but this differed to the 'real-life' study context desired by senior investigators, where supervision of CHWs would not be available.	Regular communication between field team and senior investigators helped raise ongoing issues with turnover and recruitment of CHWs in project areas.		Long-established working relationship with field coordinator with scientific and practical experience was highly valued by senior investigators unable to get to the study sites regularly due to security concerns.		Presence of study registrar in health facilities occasionally resulted in blurring of the lines between research and clinical practice, when asked to assist with clinical care.
**9**	Some difference in interpretation of the role of field workers conducting exit interviews, with some perceiving a role in (clinically) assessing the appropriateness of the patients' diagnosis and prescribed treatment.		Discussions with qualitative research team led to awareness of health workers' suspicion of the accuracy of the RDTs, leading to a decision to develop a re-education session.			Possible overlap identified in health workers' perceptions of monthly collection of routine data by field workers as supervision of their practice.
**15**	Some health workers' perceptions of the value of the trial impacted on their motivation to recruit patients to the trial.	Regular team communication enabled discussion about how to deal with health workers' questions about responding to negative RDT test results.		Locally-situated senior investigators with coordinators in study sites helped identify other research commencing in the area and to reflect on its potential impact for the study.	Fieldworkers noted in field diaries the replacement of a health worker at one study site, which proved valuable at the analysis stage for interpreting the lower rate of recruitment of patients to the study by this health worker.	

[1] Study numbers correspond to the identification numbers allocated through the ACT Consortium: www.actconsortium.org

[2] CMDs - community medicine distributors; [3] DSVs - drug shop vendors; [4] PI – principal investigator; [5] RDTs - rapid diagnostic tests (for malaria); [6] CHWs - community health workers
